# Imaging and Clinicopathological Features of Acinar Cell Carcinoma

**DOI:** 10.3389/fonc.2022.888679

**Published:** 2022-06-07

**Authors:** Qianqian Qu, Yinghui Xin, Yifan Xu, Yao Yuan, Kai Deng

**Affiliations:** ^1^ Department of Radiology, The First Affiliated Hospital of Shandong First Medical University (Shandong Provincial Qianfoshan Hospital), Jinan, China; ^2^ Department of Radiology, Shandong Provincial Hospital Affiliated to Shandong First Medical University, Jinan, China; ^3^ Department of Radiology, Qilu Hospital of Shandong University, Jinan, China

**Keywords:** acinar cell carcinoma, imaging features, clinicopathological features, treatment, prognosis

## Abstract

**Background:**

Acinar cell carcinoma (ACC) is a rare pancreatic epithelial malignancy that poses a significant threat. However, there are few related clinical studies. The present study aimed to analyze the imaging and pathological features of ACC to provide a reference for better diagnosis and treatment planning.

**Methods:**

Thirty-nine with ACC, referred to Qianfoshan Hospital, Qilu Hospital and Provincial Hospital in Shandong Province from December 2012 to December 2020, were enrolled. Their imaging and clinicopathological features were analyzed. They were followed up for 1 year, and Cox regression was used to analyze the factors affecting patient prognosis.

**Results:**

ACC was more common in the middle-aged and elderly and peaked at approximately 60 years. The clinical manifestations of the patients were mostly flatulence and upper abdomen pain. The tumor was located in the head of the pancreas in 19 cases, with an average size of 5.8 cm. We found nerve invasion and liver metastasis in one case each. 8 patients showed irregular amorphous tumor calcification on plain computed tomography and 5 showed high and low signals on T1- and T2-weighted images, respectively. Immunohistochemistry revealed 100.0% positive rates for CK, β-catenin, and Ki-67. Thirty-three patients underwent surgical resection, and the 2-year overall mortality rate was 25.6%. Cox analysis revealed that smoking was an independent risk factor affecting patient prognosis.

**Conclusion:**

An in-depth understanding of the imaging and clinicopathological features of ACC is conducive to better diagnosis and treatment planning for ACC and subsequent improvement in patient prognosis.

## Introduction

Acinar cell carcinoma (ACC) is a rare pancreatic epithelial malignancy derived mainly from pancreatic acinar cells and terminal branches of the pancreatic duct, accounting for approximately 1% of all pancreatic tumors (1). The hallmark pathological feature of ACC is its exocrine function and its potent capacity to invade and metastasize (2), which makes treatment of ACC more difficult than that of other pancreatic tumors and leads to an extremely pessimistic prognosis (3). The 5-year mortality rate of patients with ACC exceeds 50%, and its lethality ranks among the highest among all malignancies (4). However, due to the rarity of ACC and its different morphological characteristics, research on ACC is not homogeneous at home and abroad, and its diagnosis remains controversial (5). In clinical practice, ACC can only be confirmed by surgery or biopsy, which has a great hidden peril for its treatment (6).

Although ACC falls into the category of pancreatic tumors, its pathological manifestations differ from those of conventional pancreatic cancer. Therefore, comprehensive clinical practice guidelines are needed for its differentiation (7). Currently, imaging remains one of the best methods with high accuracy for early diagnosis of tumors (8). ACC, due to acinar secretion, shows substantial cystic changes on imaging (9), which may be the key to early diagnosis of ACC. Confronted with the deficiency in the current clinical research on ACC, further understanding of ACC-related lesions is the basis for improving the diagnosis rate and ensuring patients’ life and health. In this study, the imaging and pathological features of ACC patients confirmed by pathology in our hospital were analyzed, with an aim of improving the clinical awareness regarding ACC and providing a reference for better diagnosis and treatment planning.

## Materials and Methods

### Patient Information

Thirty-nine patients with ACC, admitted to Qianfoshan Hospital、Qilu Hospital and Provincial Hospital in Shandong Province between December 2012 and December 2020, were selected for this retrospective analysis. Of them, 23 patients had undergone computed tomography (CT) and magnetic resonance imaging (MRI) examinations and 16 had undergone only CT examination. The study design was approved by the institutional ethics committee.

### Eligibility Criteria

Patients aged > 18 years, diagnosed with ACC by surgery or pathological puncture, and having complete case data, were enrolled. In contrast, those with multiple tumors, cardiovascular and cerebrovascular diseases, autoimmune deficiency, organ dysfunction, or history of surgery, radiotherapy, and chemotherapy were excluded. In addition, pregnant or lactating patients and those who received antibiotic treatment within half a year before admission or had a life expectancy < 1 month were also excluded.

## Methods

### CT Examination

15 patients underwent CT using the following GE Discovery CT750HD scan parameters: voltage, 120 kV; current, 105-524mA; layer thickness, 5 mm; and spacing, 1 mm. 15 patients underwent CT using the following GE Discovery CT750 HD and Philips Brilliance iCT.The remaining 9 patients underwent CT using the following Toshiba Aquilion ONE 320 Slice CT scan parameters: voltage, 120 KV; current, 120mA; slice thickness, 5 mm; pitch, 0.7; rotation time, 0.5 s; and scanning time, 8.6 s. The scanning range for all patients was from the parietal septum to the level of the anterior superior iliac spine. The plain and contrast-enhanced CT scans were reconstructed by 1.25 mm and transmitted to the PACS software. After plain scanning, 15patients were injected with 2mL/kg of iopromide contrast agent (Bayer, Germany) at a rate of 2.5-4 mL/s, 24 patients underwent forearm vein injection of 80 ml iohexol (350 mgI/ml) with a 2.8ml/s injection rate, and 10 ml physiological saline was injected at the same rate; arterial, portal, and delayed phase scans were performed at 30, 65, and 120 s after injection, respectively.

### MRI Examination

14 of the 39 patients underwent MRI. Using 3.0T vero MR scanner (Simens, Vero, Germany) with the body coil; axial T1WI repetition time, 1000 ms; echo time, 5.6ms; T2WI repetition time, 1400 ms; echo time, 92 ms; T2WI repetition time of fat pressing, 4820 ms; echo time, 83 ms; field of view, 400 mm×400 mm; matrix 320×320, collected twice; slice thickness, 5 mm; and spacing 1 mm. 9 patients using 3.0T vero MR scanner (GE,HDX TWINSP); axial T1WI repetition time, 800; echo time, 6.9 ms; T2WI repetition time of fat pressing, 4500 ms; echo time, 85ms; Field of view (FOV), 380mm×380 mm; matrix 320×320, collected twice; slice thickness, 5 mm; and spacing 1 mm.

### Pathological Examination

Immunohistochemical staining was performed on tumor tissue sections of patients to examine indexes, including those for CK, CK-7, CK-19, Synaptophysin, β-catenin, vimentin, and Ki-67.

### Evaluation Criterion

The primary endpoints were lesion site and morphology, tumor diameter line, tumor capsule condition, fat encapsulation, bleeding and calcification, cystic degeneration (necrosis), and plain and enhanced CT values of solid components of the lesion. All images were evaluated by two senior radiographers, and the consensus reached by them was considered the examination result. The histopathological results were evaluated by senior pathologists in our hospital, and the histological sections were staged according to the American Joint Committee on Cancer staging system.

### Follow-Up for Prognosis

All patients were followed up for 1 year through hospital reexamination.

### Statistical Methods

The statistical software used for data analysis and processing was SPSS22.0. Categorical variables are expressed as percentages and compared using the chi-square test. Continuous variables are presented as mean ± standard deviation, and the comparison was made by independent sample t-test, as well as one-way ANOVA and LSD *post hoc* test. Cox regression analysis was used to determine the related influencing factors. Differences were considered statistically significant when P<0.05.

## Results

### Pathological Changes

Tumor location: The tumor was located in the head of the pancreas in 48.7% (n=19) of the cases, in the body of the pancreas in 25.6% (n=10) and in the tail of the pancreas in 25.6% (n=10).

Size: According to the imaging results, the tumor size ranged from 1.48 to 13.2 cm, with an average of 5.77 cm.

Capsule: 28 cases with an intact capsule showed focal invasion of peripheral pancreatic tissue, while in 11 cases, the lesion broke through the capsule without a clear boundary or complete capsule.

Morphology: The tumors were round or quasi-round in 31 cases and lobulated or irregular in 8 cases.

Central density: It was observed that the tumors in 79.49% (n=31) patients had varying degrees of central low density on enhanced CT images with a density of <50%, and the tumors in 20.51% (n=8) patients were cystic lesions.

**Figure 1:**
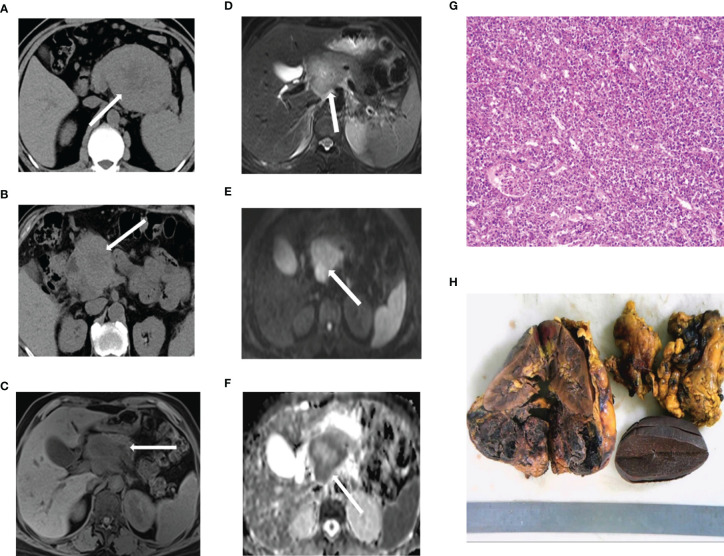
Preliminary computed tomography and magnetic resonance imaging findings **(A)** ACC in the tail of the pancreas. **(B)** ACC in the head of the pancreas. **(C–F)** ACC in a 66-year-old man. T1-weighted **(C)** and T2-weighted **(D)** images reveal a well-defined cystic lesion in the pancreatic head indicated with an arrow. Diffusion-weighted imaging **(E)** shows the area of high signal, and ADC **(F)** reveals, on the contrary. **(G)** Immunohistochemical staining of the tumor cells. **(H)** This specimen is of a 7.5-cm, well-encapsulated mass obtained from the pancreatic tail through the Whipple resection. ACC, acinar cell carcinoma.

Other pathological changes: We observed nerve invasion in 1 case and liver metastasis in 1 case, without vascular invasion or lymph node metastasis (**Figure 1**).

### Clinical Manifestations

The clinical manifestations of the patients were mostly flatulence and pain in the upper abdomen. Further, 17.95% (n=7) patients had jaundice and 12.82% (n=5) felt a significant mass pressing the abdomen. Tumor marker examination showed that CEA and CA199 levels were all within the normal range, while elevated AFP levels were found in 12.82% (n=5) patients. None of the patients developed joint disease or subcutaneous fat necrosis.

### Calcification and Bleeding

**Figure 2:**
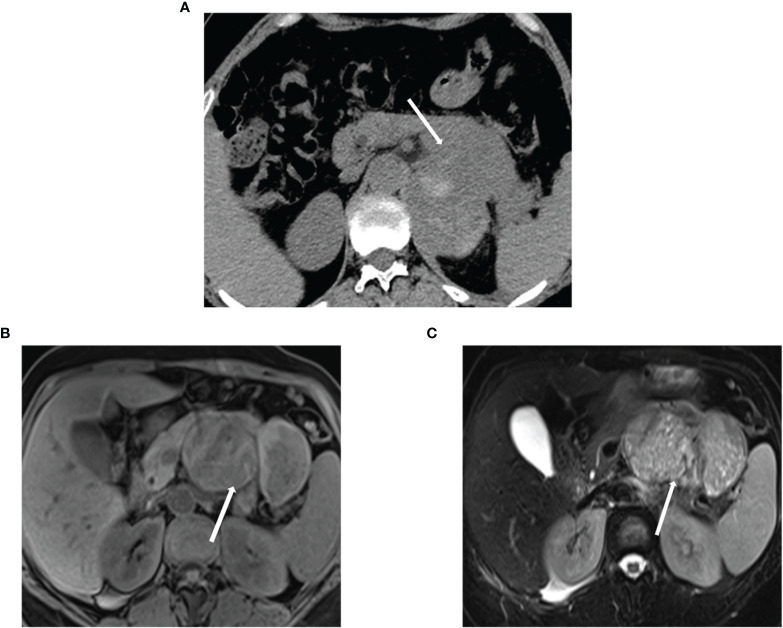
Calcification, bleeding, and necrosis **(A)** ACC in a 67-year-old woman. Unenhanced CT image reveals a pancreatic mass with irregular punctual calcifications (arrow). **(B, C)** T1-weighted and T2-weighted images reveal the tumor with the cystic necrosis (arrow). ACC, acinar cell carcinoma.

Of the patients, 20.51% (n=8) showed irregular amorphous tumor calcification on plain CT scans, and 12.82% (n=5) showed high signal on T1-weighted images and low signal on T2-weighted images. A total 20.51% (n=8) patients showed an unevenly high signal on T1 and T2 weighted images but no high density on plain CT scans (**Figure 2**).

### Dynamic Enhanced Scanning

**Figure 3:**
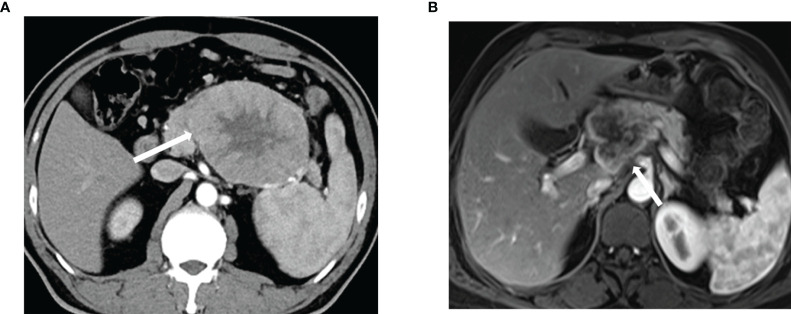
Biphasic contrast enhanced computed tomography and magnetic resonance imaging of ACC **(A)** Venous enhancement is heterogeneous. **(B)** There is uneven enhancement of lesions in the portal arterial phase. ACC, acinar cell carcinoma.

Arterial CT and MRI results showed uneven enhancement for all lesions except a cystic tumor, the enhancement of which was lower than that of the surrounding normal pancreas, and no enhancement was observed in the cystic necrosis area (**Figure 3**).

### Duct Distribution

**Figure 4:**
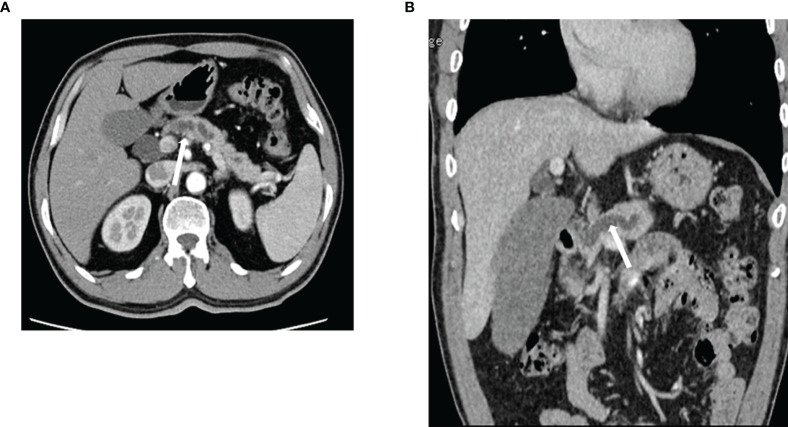
Expansion of the pancreatic duct. ACC in a 63-year-old man **(A, B)** Contrast-enhanced computed tomography reveals ductal dilatation. ACC, acinar cell carcinoma.

Among the 19 patients with pancreatic head tumors, 26.31% (n=5) showed mild dilatation of the bile duct tree and main pancreatic duct and 52.63% (n=10) showed mild dilatation (**Figure 4**).

### Surgical Treatment and Tumor Metastasis

A total of 84.61% (n=33) patients underwent surgical resection after admission, including 20 patients who underwent pancreaticoduodenectomy and 13 patients who underwent distal pancreatectomy. A total of 17.95% (n=7) patients developed lymph node metastasis, including 2 case of liver invasion, 4 cases of duodenal invasion, and 1 case of bile duct invasion. All patients received palliative chemotherapy (8 to 12 cycles of capecitabine combined with oxaliplatin (XELOX) after admission.

### Immunohistochemical Results

**Figure 5:**
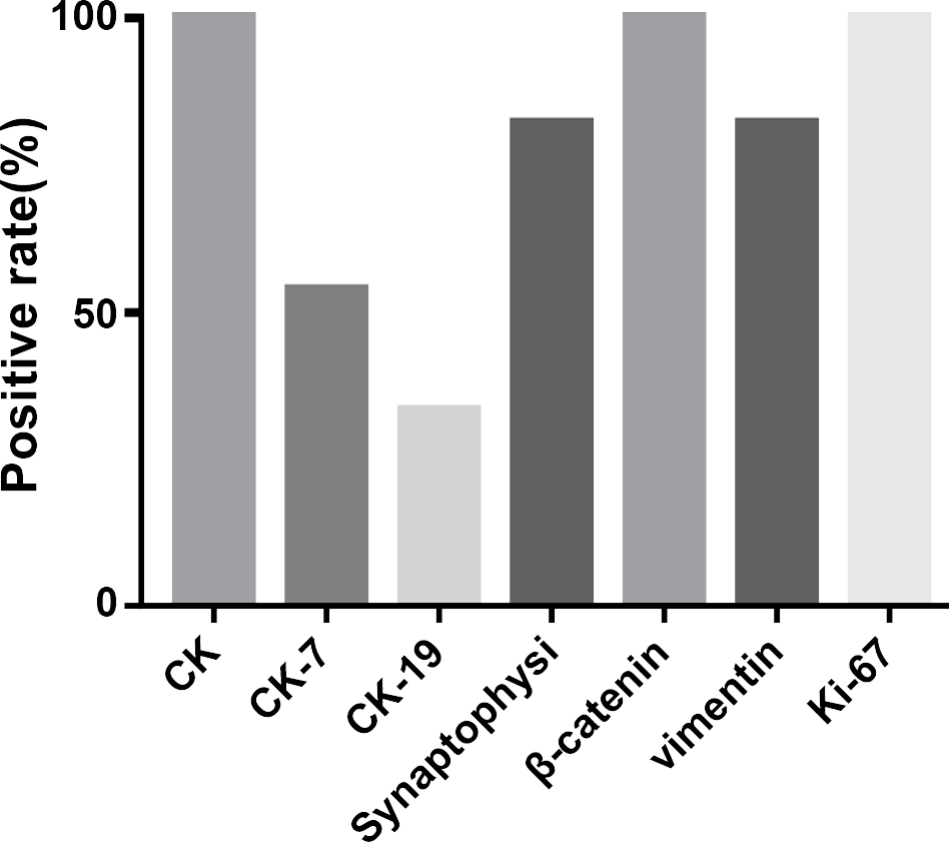
Immunohistochemical results.

Immunohistochemistry revealed 100.0% positive rates for CK, β-catenin, and Ki-67. The positive rates were 82.1% for both Synaptophysin and Vimentin, 53.8% for CK-7, and 33.3% for CK-19 in patients with ACC (**Figure 5**).

### Prognostic Follow-Up

34 of the 39 patients were successfully followed up, of which 10 patients died, with a 2-year overall mortality rate of 25.6%. COX analysis revealed that smoking was an independent risk factor affecting the prognosis of patients with ACC (P<0.05) (**Table 1**).

**Table 1:** Cox analysis of factors influencing the prognosis of patients with acinar cell carcinoma.

	Univariate analysis			Multivariate analysis		
	RR	95% CI	P	RR	95% CI	P
Age	1.608	0.842-2.942	0.124	–	–	–
BMI (kg/cm^2^)	1.184	0.642-2.542	0.541	–	–	–
Gender	2.962	1.242-4.842	0.321	–	–	–
Family medical history	0.658	0.242-4.523	0.207	–	–	–
Smoking	2.064	1.421-3.604	0.012	2.354	1.242-4.641	0.004
Past pancreatic diseases	1.608	0.842-2.942	0.124	–	–	–
Clinical presentations	1.184	0.642-2.542	0.541	–	–	–
Lesion size	3.542	0.684-8.612	0.292	–	–	–

BMI, body mass index; CI, confidence interval; RR, P.

## Discussion

ACC, an extremely rare type of pancreatic cancer, is highly harmful and has a dire patient prognosis, albeit with a low incidence rate (10, 11). ACC mainly manifests as pancreatic lesions. When tumors invade the surrounding organs, large space-occupying lesions can be formed in metastatic organs, which may easily lead to misdiagnosis as other neoplastic diseases during preoperative imaging (12). Second, the early clinical manifestations of ACC are usually abdominal pain and bloating, without other pathological functional changes, which is one of the key reasons ACC is initially ignored (13). Improving the clinical examination protocol of ACC is imperative to ensure patient safety and improve prognosis. In this study, the imaging and clinicopathological features of ACC were preliminarily analyzed, and the results are as follows.

### Clinical Features of ACC

According to the clinical data of the study participants, ACC is more common in the middle-aged and elderly, and peaks at about 60 years, which is consistent with previous findings (14) and can thus prove the accuracy of our experiment. In addition, male patients are slightly more than female patients. There is no obvious familial inheritance, but the disease may be connected with a history of pancreatic diseases. Abdominal pain is the main clinical manifestation, with no other remarkable clinical symptoms. Routine tumor marker examination shows no significant changes in CEA and CA199 levels. In addition, according to the literature reports, CA125 is closely related to ACC progression, elevation of CA125 in CAA is common clinically, but it is also associated with digestive tumors. Further studies are required to confirm the relationship between them.

### Pathological Features of ACC

Compared with other pancreatic tumors, ACC usually presents as larger tumors with expansive growth which and clearer margins at onset. The tumor capsule is mostly solid. Cystic degeneration, necrosis, and hemorrhage are commonly observed at the center of large lesions. Microscopically, ACC presents a relatively dense cellular structure separated by fibrous stroma; the cells have abundant cytoplasm, visible proeosinophilic granules, and show rapid mitosis. It has been suggested that immunohistochemical labeling of pancreatic enzyme products could improve diagnosis of ACC (15), which may be related to our findings. However, Chou et al. observed that ACC-labeled trypsin was positive and chymotrypsin and lipase were also highly sensitive (16), which is consistent with the results of this study. In addition, ACC shows an obvious partial endocrine differentiation tendency, and some lesions are positive for chromogranin and synaptophysin markers.

### Imaging Features of ACC

The shape of the ACC is mostly irregular and grows along the long axis of the pancreas. It compresses the surrounding tissues and causes collagen fibers to proliferate to form a pseudocapsule with relatively clear boundaries. However, due to the slow growth of tumors and lack of neurophilic sites, most tumors lack blood supply, and the arterial phase enhancement is lower than that of the pancreatic tissue. However, the tumor contains almost all sinusoids, showing progressive enhancement. The higher the tumor differentiation, the smaller the volume, the more uniform the blood supply, and lesser the area of necrosis. Both CT and MRI manifestations show a uniform honey degree, while T1WI and T2WI show slightly higher signals, and the enhancement presents a gradual increase. The tumor is poorly differentiated, large, and abundant calcification, hemorrhage, and necrosis can be seen when the blood supply is unbalanced. CT and MRI show mixed density and signals. The larger the range of hemorrhagic necrosis, the more uneven the enhancement. MRI is more effective for the inspection of intratumoral hemorrhage, and CT is more effective for the inspection of calcification. According to the results of this experiment, we can summarize the imaging features of ACC as follows: 1. Larger mass. The average lesion size was 5.8 cm. It was <2 cm in only one case, and the maximum size observed was 22 cm. This is consistent with the findings of previous studies (17). 2. The disease may occur anywhere in the pancreas, most frequently in the head of the pancreas. 3. The lesion has clear boundaries. Contrast-enhanced scanning shows linear enhancement of the capsule in some lesions, but in most of them it is incomplete, with local invasion of adjacent tissues. The capsule can be seen in most lesions, which is also consistent with previous research results (18). 4. Rare calcification. 5. The tumors mainly have solid components: with different proportions of low-density regions, this area was not significantly enhanced and indicated hemorrhage or necrotic cystic degeneration. 6. ACC is mostly a hypovascular lesion (19), the enhancement degree of which is lower than that of the adjacent normal pancreatic parenchyma in each phase, and the arterial phase enhancement is greater than that of the normal pancreatic parenchyma.

### Differential Diagnosis of ACC

Combining our results with those of previous studies, we summarize the differential diagnosis of ACC. ACC has larger lesions, sharper margins, and earlier enhancement peaks than pancreatic ductal adenocarcinoma, the most common form of pancreatic cancer. The tissue structure of ACC is similar to that of pancreatic neuroendocrine tumor (20), with consistent positive rates of some labeled proteins (21). However, pancreatic neuroendocrine tumors are characterized by cystic degeneration and necrosis accompanied by hemorrhage, and most cases have a malignant tendency, ill-defined boundaries, dilation of the pancreatic duct, and metastasis (22). The clinical manifestations of solid pseudopapillary tumors are quite different. Solid pseudopapilloma of the pancreas is more common in young and middle-aged women, especially in the tail of the pancreas (23); although the capsule is equally common, calcification is also significant (24).

### Prognostic Factors Affecting ACC

As we all know, the meaning of independent risk factors is that the larger the lesion, the higher the risk of death for the patient’s prognosis. If RR<1, it is a protective factor (that is, the larger the value, the lower the patient’s prognostic risk). This suggests that the risk of death in our patients was mainly affected by disease progression. In other words, early diagnosis of ACC can effectively improve the prognosis and survival rate of patients, which was the focus of this study.

### Limitations of This Study

First, to ensure the uniformity, we followed up all patients for one year. However, this prevented evaluation of the relationship between imaging findings and the long-term prognosis of patients. Therefore, a longer follow-up investigation is necessary. Moreover, since ACC is relatively rare, its clinical treatment requires improvement. Third, it is unclear if different treatment modalities are key to determining the prognosis of patients. Last, but not least, because ACC is rare, MRI multifunctional imaging was not available earlier. In addition, this study did not explore the characteristic changes, including lipase hypersecretion syndrome, multiple subcutaneous fat necrosis, and eosinophilia. To ensure that the results were representative, the patient data included typical imaging manifestations of solid pancreatic cystic lesions. This also resulted in a sample size of only 39, which was too small to evaluate the aforementioned characteristic changes. In this study, only 23 patients underwent MRI. Therefore, in future, a combination of preoperative diagnosis, pathology, and MRI must be used for ACC diagnosis. We will continue to deepen the relevant research on ACC to obtain more accurate findings for clinical reference. As an important immunohistochemical data, BCL-10 was not included in this manuscript due to the limitations of objective reasons. We realized that BCL-10 combined with imaging examination might provide more diagnostic information, which provided a direction for our future research.

In summary, ACC of the pancreas is a rare tumor. MRI and CT are complementary imaging methods. CT is sensitive to central calcifications. MRI is superior for observing the relationship between lesions and normal tissues, components of lesions, internal hemorrhage of tumors, and ductal dilatation, among other factors. Despite the rarity of this disease, further imaging and pathology based studies should be performed to determine the clinical findings and treatment outcomes of this disease.

## Author Contributions

All authors listed have made a substantial, direct, and intellectual contribution to the work and approved it for publication.

## Conflict of Interest

The authors declare that the research was conducted in the absence of any commercial or financial relationships that could be construed as a potential conflict of interest.

## Publisher’s Note

All claims expressed in this article are solely those of the authors and do not necessarily represent those of their affiliated organizations, or those of the publisher, the editors and the reviewers. Any product that may be evaluated in this article, or claim that may be made by its manufacturer, is not guaranteed or endorsed by the publisher.
